# Editorial

**DOI:** 10.4102/sajhivmed.v21i1.1199

**Published:** 2020-12-21

**Authors:** David C. Spencer

**Affiliations:** 1 Division of Infectious Diseases, Department of Medicine, Helen Joseph Hospital, University of the Witwatersrand, Johannesburg, South Africa

Greetings to all readers of the *Southern African Journal of HIV Medicine* (SAJHIVMED) and members of the Southern African HIV Clinicians’ Society!

This has been a long year and one that will not be forgotten easily. Coronavirus disease 2019 (COVID-19) has reshaped our world.

Thank you to our nurses and doctors who stand by the bedside of the sick, placing life and future on the line for others. You have done a great job, and we are proud of you. Thank you too, to researchers, laboratory staff and industry colleagues for your backroom work. Without the goodwill of all, the sick are easily abandoned. Thankfully, effective vaccines are emerging. But the pandemic is not over. Will low- and middle-income countries (LMICs) access these therapeutics? Time will tell. Clearly, this is an issue that we will closely watch.

Thank you to our authors. Your vision of human immunodeficiency virus (HIV) in Africa is important to us: current, authentic and compelling. You have allowed science/truth to do the talking. Thank you for your submissions. Articles are already being allocated to our 2021 edition! If you too have HIV-related research based in LMICs, especially in Africa, please consider submitting to the SAJHIVMED.

Thank you to our reviewers. Thank you for saying yes. For returning reviews on time and ensuring we meet international publication standards and timelines! You are the backbone of our success and we are very much in your debt.

If you are taking a break over the December holidays, we invite our readers to do some catch-up journal reading. This year we have shared 60 articles with you. The majority were the product of ‘original’ research. A small number included reviews, case reports and letters. Five are new guidelines. I will restrict my comments to these. But please do not forget the remaining 55 articles. These papers reflect this changing epidemic on our doorstep. Stay up to date.

The *2020 Updated ART guideline* is essential reading.^1^ This is high-end contemporary science with a strong clinical appeal. A lot about dolutegravir. But with many ‘insights’ and ‘tips’ not found elsewhere. A very special guideline, it deserves your attention.

Dr Jeremy Nel and his co-authors have also given us an African ‘first’, the *guidelines for solid-organ transplantation in persons living with HIV (PLWH)*.^2^ This is a collaboration of local and international HIV clinicians and senior transplant surgeons. Relevant to Africa? Yes. Transplant organs are needed by HIV-infected and uninfected all over Africa. This article rewards the reader with accessible immunology and a practical approach to complex patient care.

Human immunodeficiency virus-prevention is addressed in two guidelines. *The updated South African National Guideline for the Prevention of Mother to Child Transmission of Communicable Infections (2019)* and the *Southern African Guideline on the safe, easy and effective use of Pre-exposure Prophylaxis (PrEP): 2020*.^3,4^ The former is an update of the 2019 National prevention of mother-to-child transmission (PMTCT) guidelines. Its underlying concern is the relative increase in viral transmission in the first 18 months of life: 0.7% at birth (2019) but 4.3% at 18 months (2016). (More recent 18-month data are clearly needed). Quo vadis re. breastfeeding? Children in Africa remain at risk of HIV infection. These guidelines set out the rules, the goalposts. Please read and implement these guidelines.

This is an extraordinary report.^5^ The authors comment, ‘To our knowledge, no PMTCT data of this *magnitude* has been published from a low-income, high HIV-prevalence setting’ before! This is a 14-year review of the PMTCT in a large cohort of Sowetan women and their children. Over this period 360,751 pregnant women were managed in 13 clinics in Soweto, South Africa. The proportion of pregnant women living with HIV who attended these clinics rose from 14.3% in 2009 to 45% in 2015 (*p* < 0.001)! Prevalence rates of HIV in pregnancy during the period were high: 28.9% in 2002, 33.1% in 2009 and 27.4% in 2015. Nevertheless, the clinic staff clinics and the project funders -overseas and local (SA government) – managed to reduce the mother-to-child transmission rate at 6 weeks of life from 7.0% in 2007 to < 1.6% in 2013-2015, *p* < 0.001. The report is astonishing. The South Africa of recent decades has not been easy to live in particularly for the poor. More so for those living with HIV. The authors acknowledge limitations to their work. These do not detract from the body of the work or lessen its impact.

Prof. Bekker’s ‘safe, easy and effective use of PrEP’ paper comes on the heels of data confirming the superior efficacy of two-monthly injections of cabotegravir as against oral long-term PrEP in 3223 African women (Study HPTN 084) and men who have sex with men (MSM) and transgender women (Study HPTN 083). https://clinicaltrials.gov/ct2/show/NCT03164564. (HTPN 084), https://clinicaltrials.gov/ct2/show/NCT02720094. (HTPN 083).

Human immunodeficiency virus prevention is of critical importance to South Africa (SA). The current population of SA is ± 59.62 million; *± 7.8 million are PLWH and ± 18.7% of our 15–49-year-olds are living with HIV*.^6^ I recommend this PrEP guideline to every healthcare worker. These numbers demand action. Although only *oral* PrEP is available in SA at this time, injectables will come. We need to get young people in particular thinking new thoughts about their sexual health. A companion article speaks to the ethics and legalities of adolescent PrEP.^7^ This is particularly helpful for family physicians and parents who wish to protect sexually active children.

The fifth guideline has broad application in sub-Saharan Africa. *The Southern African HIV Clinicians’ Society guideline for harm reduction* focuses on drug addiction in PLWH.^8^ Justice Edwin Cameron provides a wise and considered response. Please read it.^9^ The guideline is long but comprehensive. The writers are experts in the field. Throughout, a human rights understanding is followed and a compassionate yet evidence-based approach to illicit drug use is outlined. Addiction is increasing in Africa. Many also live with HIV. New thoughts and new approaches are needed. This is a timely but sober read. If you care for your patients, do not sidestep the issue.

I have left you with another 55 articles to explore in the days ahead. Many of these can stand with the best in the field. Take a look at this year’s volume.

This year marks the 20th anniversary of the ‘birth’ of the *Southern African Journal of HIV Medicine*.^10^ It was the brainchild of the then president-elect of the Society, Prof. Des Martin, and others on the executive committee. We published the first issue to coincide with the 13th International HIV Conference in Durban (SA) in July of 2000. The first journal is a brief read but provided colleagues and the South African nation with its first ever ‘national’ antiretroviral (ART) guideline. Thank you, Des. Thank you, colleagues. We have come a long way since then. The lives of thousands have been rescued. Words cannot measure the success.
